# COVID-19 pandemic–related adaptations of medical education in clinical pharmacology — impact on students and lecturers at a German university

**DOI:** 10.1007/s00210-022-02225-3

**Published:** 2022-03-18

**Authors:** Susanne Hafner, Oliver Zolk, Holger Barth

**Affiliations:** 1 Institut für Naturheilkunde und Klinische Pharmakologie, Universitätsklinikum Ulm, 89081 Ulm, Germany; 2 Institut für Klinische Pharmakologie, Medizinische Hochschule Brandenburg Theodor Fontane, Immanuel Klinik Rüdersdorf, 15562 Rüdersdorf, Germany; 3 Institut für Pharmakologie und Toxikologie, Universitätsklinikum Ulm, 89081 Ulm, Germany

**Keywords:** COVID-19 pandemic, Education, e-learning, Pharmacology, Attitude, Survey

## Abstract

In medical studies, pharmacology is a subject with a high teaching and study load. The COVID-19 pandemic–related switch to distance learning implies challenges for students and teachers. To identify changes of behavior, attitudes, and needs among students and teachers in clinical pharmacology during the pandemic, regular surveys were conducted at Ulm University, Germany. Overall, 884 students and 5 teachers answered the survey. Over time, students’ usage of learning materials in print form and further literature (textbooks, guidelines) constantly decreased. In turn, most students used digital materials provided via a learning management system. Attitudes and opinions of students and teachers significantly differed regarding (i) the benefit of certain teaching formats and learning materials, (ii) open-mindedness towards e-learning, and (iii) future visions. Most students and lecturers stated that they spent more time on study or teaching-related activities, respectively, and had less contact with fellow students or colleagues in comparison to pre-pandemic times. Furthermore, the subjective teaching load for most lecturers (80%) increased during the pandemic. To identify determinants of learning success, the survey data were correlated with students’ gradings in the written exams of clinical pharmacology. In the first online semester (summer term 2020), the use of paper-based learning scripts was correlated with better exam grades. No correlation with grade was found for any other learning materials. Age was inversely correlated with exam grading. Striking a balance between the future visions and needs of students and teachers implies a particular challenge for the educational system of the next years.

**Supplementary Information:**

The online version contains supplementary material available at 10.1007/s00210-022-02225-3.

## Introduction

In December 2019, news features reported on a novel virus, namely SARS-CoV-2, identified in Wuhan, China. At that time, however, no one could have imagined that there would be more than 5 million cumulative deaths by the end of 2021 (https://covid19.who.int/) and that the coronavirus would have a dramatic and long-lasting impact on daily life around the world just a few weeks later: The World Health Organization declared the pandemic situation on 11 March 2020. Although the worldwide spreading and severity of COVID-19 disease was evaluated differently in distinct countries, nearly 4 billion people worldwide, i.e., half of humanity, were under some form of lockdown in April 2020 (Sandford 2020). The lockdown hit nearly every aspect of social and economic life, among others the educational system. According to the global survey report on the impact of COVID-19 on higher education by the International Association of Universities (IAU), schools and higher education facilities were closed in 185 countries in April 2020, affecting 89.4% of total enrolled learners worldwide (Marinoni et al. 2020). Despite the global consensus that education must continue, preparation for the shift to online learning has been quite unequal in different parts of the world. Of note, the feasibility of online education depends on many factors including technical prerequisites such as internet access, the skills and resources of teachers and staff, and also the proportion of practical versus theoretical skills in the respective field of study. Clearly, continuing education at medical schools and training of doctors and nurses must be a top priority in times of a pandemic with short-running healthcare capabilities. In Europe, medical education has a long tradition and the very first universities to study medicine were founded here, e.g., in Bologna/Italy, Montpellier/France, and Oxford/UK. Germany is situated in central Europe and has a long history of medical education as well, with about 40 public medical universities and 80,000 enrolled medical students (https://www.studying-in-germany.org). However, prior to the COVID-19 pandemic, there were differences in the adoption of newer digital formats in teaching between European medical schools. Whereas European countries like Denmark or Sweden had high levels of digitalization in higher education at that time, at most German universities, e-learning and online education were rather practiced as optional supplements to classroom teaching, or within single “lighthouse projects,” but not current standard (Gilch et al. 2019). A cross-sectional online survey in 2012 and 2015 including mainly students of the university of Oldenburg found that university offers of digital learning and teaching formats lagged behind the demand of students (Zawacki-Richter et al. 2016). The same group performed a secondary data analysis including data from an internal teacher evaluation and revealed that both, teachers and students, used a limited number of digital tools (Bond et al. 2018).

In the middle of March 2020, the German government decided on a nationwide lockdown to contain the COVID-19 pandemic. The resulting shift to distance learning brought new challenges for both, students and academic teachers. Within the advanced semesters of medicine, clinical pharmacology with its lectures, seminars and courses, is a subject with a high teaching and study load. The aim of this survey study was to reveal pandemic-related changes in learning and teaching in clinical pharmacology, and to identify the attitudes and needs of students and lecturers at Ulm University.

## Methods

Ulm University is located in the Southwest of Germany in the federal state of Baden-Württemberg. With 10,000 students, Ulm University is rather small facilitating close interaction between academic teachers and students. The medical faculty is the biggest of three scientific faculties with about 3,500 enrolled students. The degree course in medicine takes 6 years and is completed by a state exam and the license to work as a physician. To question medical students in classes of clinical pharmacology, paper-based questionnaires were used. The items were conceived by two lecturers, both members of the Institute of Pharmacology of Natural Products and Clinical Pharmacology. The student survey covered:

i) Personal details (age, gender)
ii) The usage of specific learning media/materials (learning scripts in digital or paper form, textbooks, treatment guidelines, e-learning materials provided by the institute)
iii) The personal attitude towards e-learning (rather open-minded or rather skeptical)
iv) The personal opinion of one’s own performance in clinical pharmacology
v) Pandemic-related changes of learning behavior (more/less time spent on study-related activities, more/less learning with fellow students, more e-learning)
vi) The evaluation of the utility of different learning materials and teaching formats (learning scripts, lectures for time-independent use, scheduled lectures, medication tasks, and comprehension questions)
vii) The preferred teaching format for the future (mainly online, classroom teaching, or a mixture of both)

In the questionnaire sub-heading, participants were asked for their consent to data processing and use within the described teaching/research project. Furthermore, students were informed on the voluntariness of their participation. Coding facilitated the correlation of survey data with the examination mark of each participant as an indicator of learning success while preserving participant anonymity. The form was handed over to students at the day of the written exam in clinical pharmacology, and the students were asked to submit the completed form afterwards.

The surveys were created and primarily analyzed using EvaSys software (evasys GmbH, Konrad-Zuse-Allee 13, 21,337 Lüneburg, Germany). Further data analyses were conducted using Excel (Office 2016). Spearman correlations were calculated using Sigma plot 14.0.

University teachers were surveyed only once in late 2021. The questions covered:

i) Personal details (age, gender)
ii) The usage of specific materials/media for lesson preparation (learning scripts from former semesters, textbooks, treatment guidelines, web-based information)
iii) The personal attitude towards e-learning (rather open-minded or rather skeptical)
iv) Pandemic-related changes of teaching behavior (more/less time spent on teaching-related activities, more/less screen work, more/less contact with colleagues)
v) The evaluation of the utility of different learning materials and teaching formats (learning scripts, lectures for time-independent use, scheduled online lectures, scheduled classroom lectures, medication tasks, and comprehension questions)
vi) The preferred teaching format for the future (mainly online, classroom teaching, or a mixture of both)

All lecturers received the survey form via email, with a short description of the project and information on the voluntariness of their participation and the anonymity of data in further processing steps. The questionnaires could be sent back via internal mail or e-mail as desired. For the small number of teachers (*n* = 8), these surveys were evaluated manually and the data were directly entered in excel tables. One of the authors participated in the teachers’ survey.

## Results

### Participants

Details on survey time and respondents’ characteristics are summarized in Table 1 for medical students and Table 2 for lecturers. Overall, 884 students answered the survey (107–237 students in each semester). Response rates were high and ranged from 79.4 to 95.2%. Participating students in the 2 more recent surveys were markedly younger than students questioned at earlier time points. This is probably due to a German school reform which was introduced in 2011/2012 and shortened secondary school education. In all semesters questioned, the majority of students were female, and the percentage of females increased over time from 56.1% in winter term 2019/2020 to 64.4% in summer term 2021. University teachers were surveyed in December 2021. Here, 5 of 8 teachers answered the survey (4 males, 1 female). Most lecturers were middle-aged (between 30 and 50 years), and only one lecturer was older than 60 (Table 2).

**Table 1:** Survey and respondents’ characteristics — students

Time	January 2020	July 2020	February 2021	April 2021	July 2021	Total/Average +/- SD
COVID-19 pandemic	Pre-pandemic	COVID-19 pandemic, no on-campus teaching at Ulm university				
Term	Winter term 2019/2020	Summer term 2020	Winter term 2020/2021	Winter term 2020/2021	Summer term 2021	
Cohort (number)	8/9	7	8/9	7	8/9	
Respondents (*n*)	107	220	155	237	165	**884**
Response rate (%)	83.6	79.4	91.7	95.2	84.2	**86.8 ± 5.8**
Sex						
Female (%)	56.1	61.1	60.1	63.9	64.4	**61.1 ± 3.0**
Male (%)	43.9	38.9	39.2	36.1	35.6	**38.7 ± 3.0**
Diverse (%)	-	-	0.7	0	0	**0.2 ± 0.3**
Age (mean, years)	24.5	23.6	24.6	22.8	22.9	**23.7 ± 0.8**
Exam grade (mean, only respondents)	-	3.2	2.9	2.3	2.5	**2.7 ± 0.4**
Exam grade (mean, all examinees)	2.72	3.31	2.92	2.34	2.95	**2.8 ± 0.3**

**Table 2:** Survey and respondents characteristics — lecturers

Time	December 2021
Respondents (*n*)	5
Response rate (%)	62.5
Sex	
Female (%)	20
Male (%)	80
Diverse (%)	-
Age (years)	
≤ 30	0
31–40	2
41–50	2
51–60	0
> 60	1

### Student’s usage of learning materials for exam preparation

**Fig. 1:**
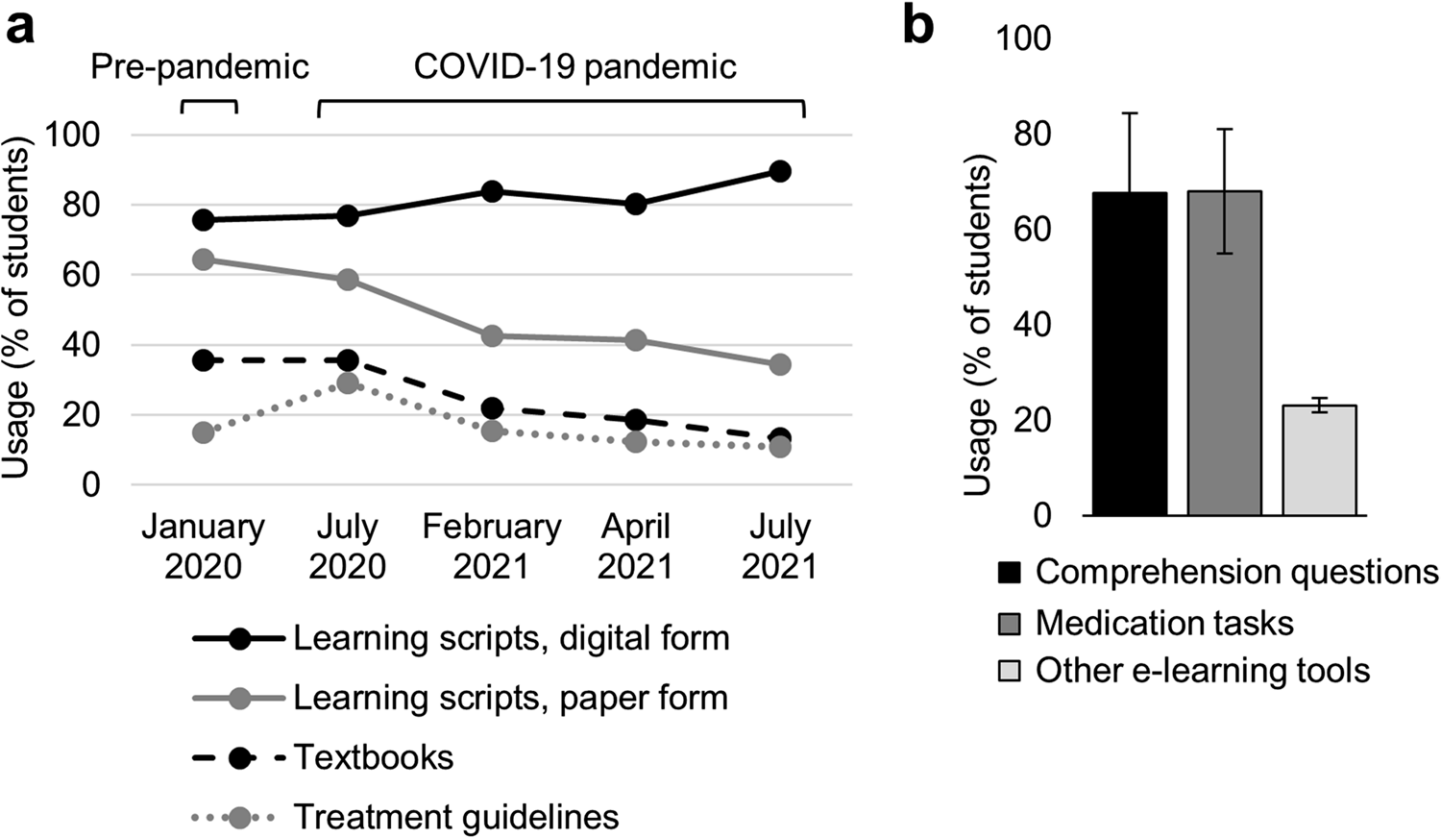
Usage of different learning materials in clinical pharmacology by medical students before and during the COVID-19 pandemic. **a** Usage of different learning materials by medical students over time (% of respondents), *n* = 884 respondents. **b** Medical students’ usage of additional e-learning offers in clinical pharmacology during the COVID-19 pandemic (% of respondents from February 2021 to July 2021), *n* = 557 respondents

Over time, students’ usage of learning materials in print form (paper-based learning scripts, textbooks) constantly decreased: Paper-based learning scripts were used by 64.5% in the pre-pandemic cohort, but only by 34.5% of students in summer term 2021 (Fig. 1a). Likewise, 35.5% indicated to learn with textbooks before the pandemic, but only 13.3% in summer 2021 (Fig. 1a). In turn, the use of digital learning scripts increased (from 75.7 to 89.7%) and most students used the e-learning tools provided by the institute consisting of medication tasks and comprehension questions (Fig. 1b). To incorporate the e-learning tools in the course design, the activities “test” and “task” provided by the learning platform were used. Table S1 shows the curriculum in clinical pharmacology at Ulm University.

### Benefit evaluation of different learning materials by students and lecturers

**Fig. 2:**
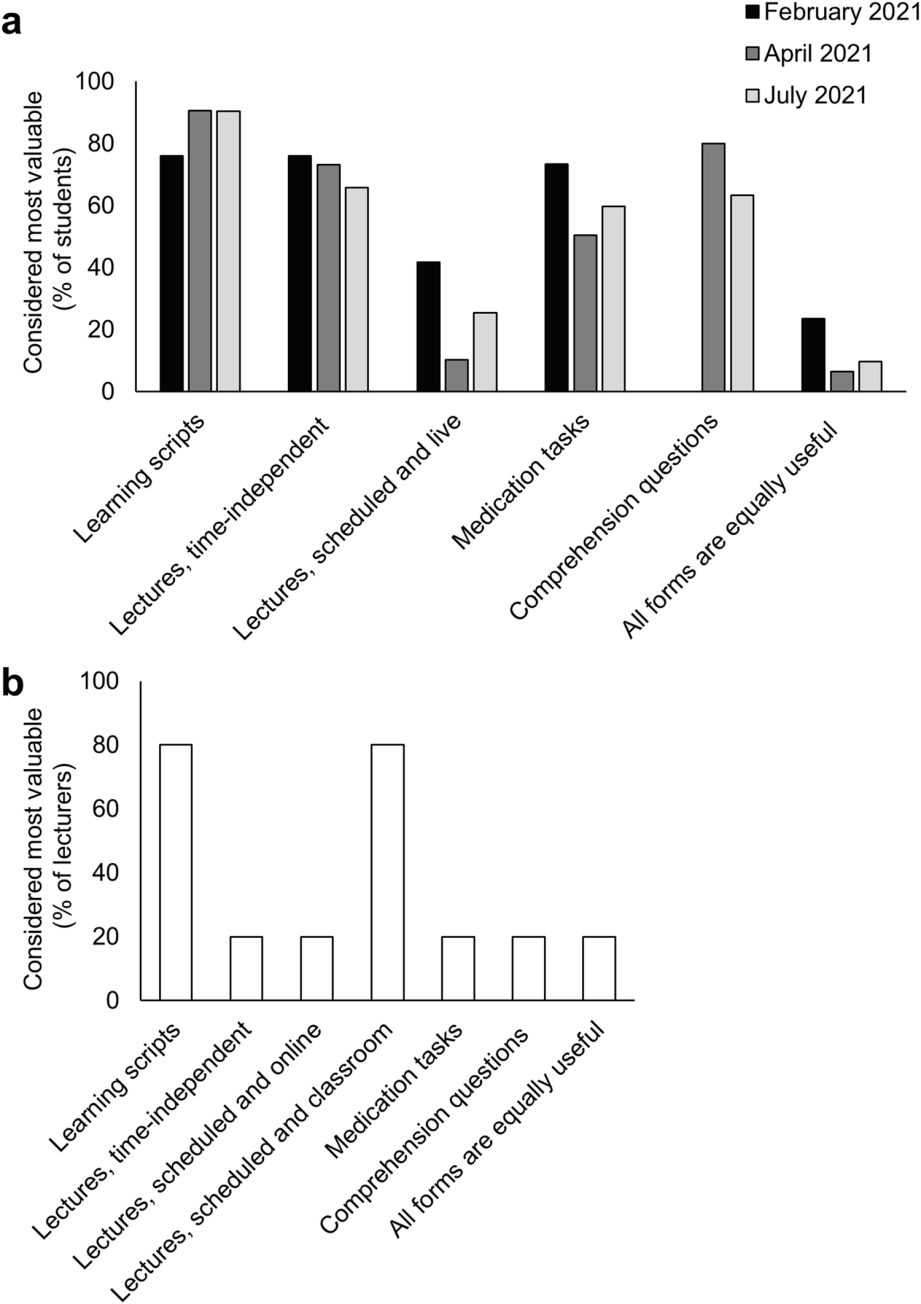
Benefit of different learning materials in clinical pharmacology evaluated by medical students and teachers during the COVID-19 pandemic. **a** Learning materials considered most valuable by medical students during the second year of the COVID-19 pandemic (% of respondents from February to July 2021), *n* = 554 respondents. **b** Learning materials considered most valuable by lecturers in the second year of the COVID-19 (December 2021), *n* = 5 respondents

Learning scripts were considered the most useful learning aid by both, students (85.7%) and teachers (80%) (Fig. 2). However, video lectures for time-independent attendance were clearly preferred over scheduled lectures by students (71.6% versus 25.7%), whereas teachers took a contrary point of view with 4 of 5 teachers considering classroom teaching as the teaching format of choice.

### Attitude towards e-learning among students and lecturers

**Fig. 3:**
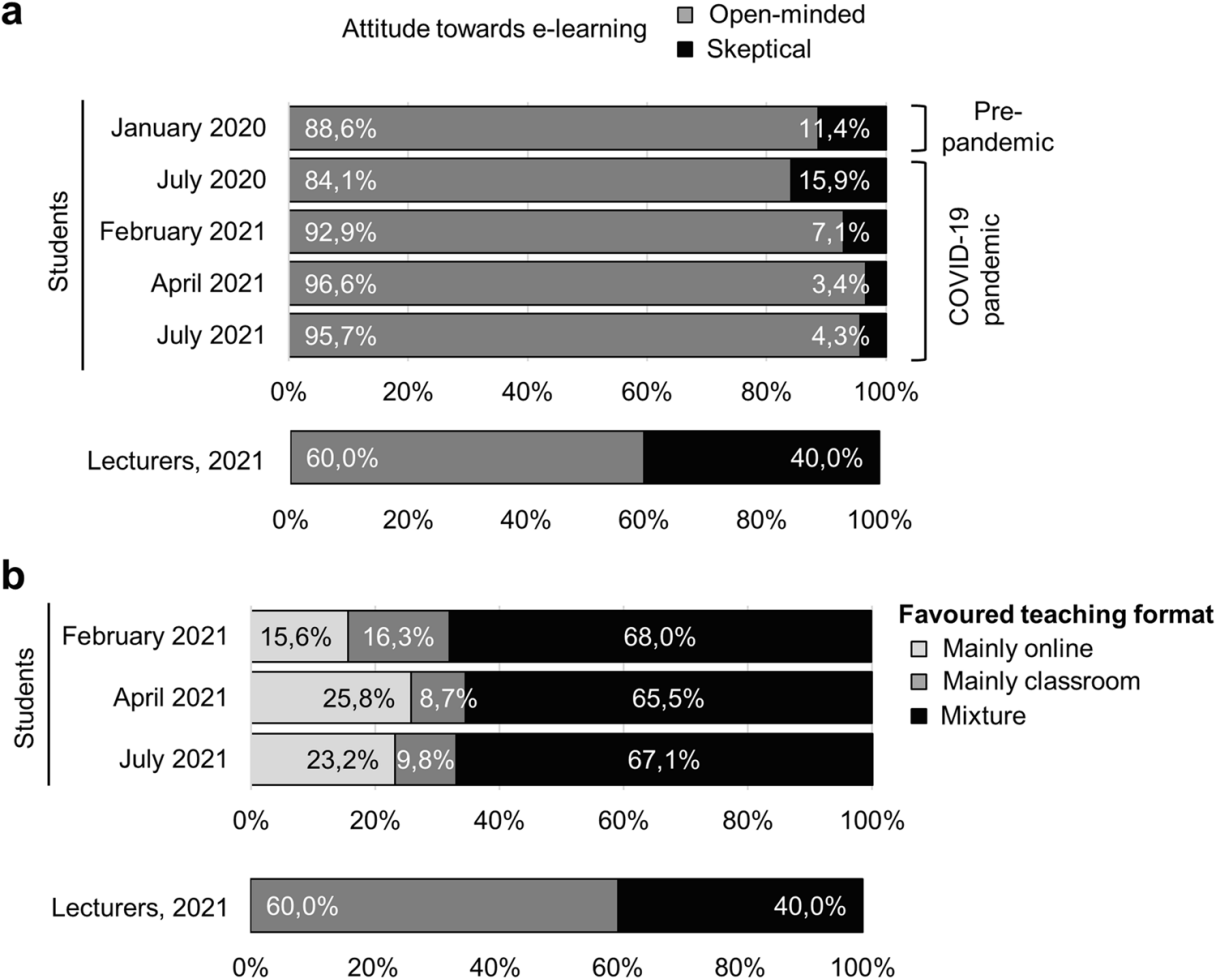
Attitude towards e-learning and preferences for future teaching among students and lecturers in clinical pharmacology. **a** Upper panel: attitude of medical students towards e-learning over time, *n* = 873 respondents. Lower panel: attitude of lecturers in clinical pharmacology towards e-learning in December 2021, *n* = 5 respondents. **b** Upper panel: students’ favorite form of teaching for the future (% of respondents, February 2021 to July 2021), *n* = 540 respondents. Lower panel: lecturers’ favorite form of teaching for the future (% of respondents, December 2021), *n* = 5 respondents

Even before the pandemic, only few students (11.4%) took a skeptical attitude towards e-learning tools (Fig. 3a). During the pandemic, e-learning skepticism further declined to < 5% in 2021. Among lecturers, a rather skeptical attitude towards e-learning was more common, with 3 of 5 teachers expressing skepticism towards e-learning even in late 2021, i.e., the end of the 2nd year of the pandemic (Fig. 3a, lower panel).

### Preferences for future teaching among students and lecturers

Preferences of students and lecturers regarding teaching formats for the future significantly differed: Whereas most students (66.8% ± 1.0%) favored a mixture of online and classroom teaching (Fig. 3b, upper panel), this was true for only 40% of the lecturers (Fig. 3b, lower panel). Among lecturers, 60% favored a return to mainly classroom teaching in the future.

### Changes of learning and teaching behavior during the COVID-19 pandemic

**Fig. 4:**
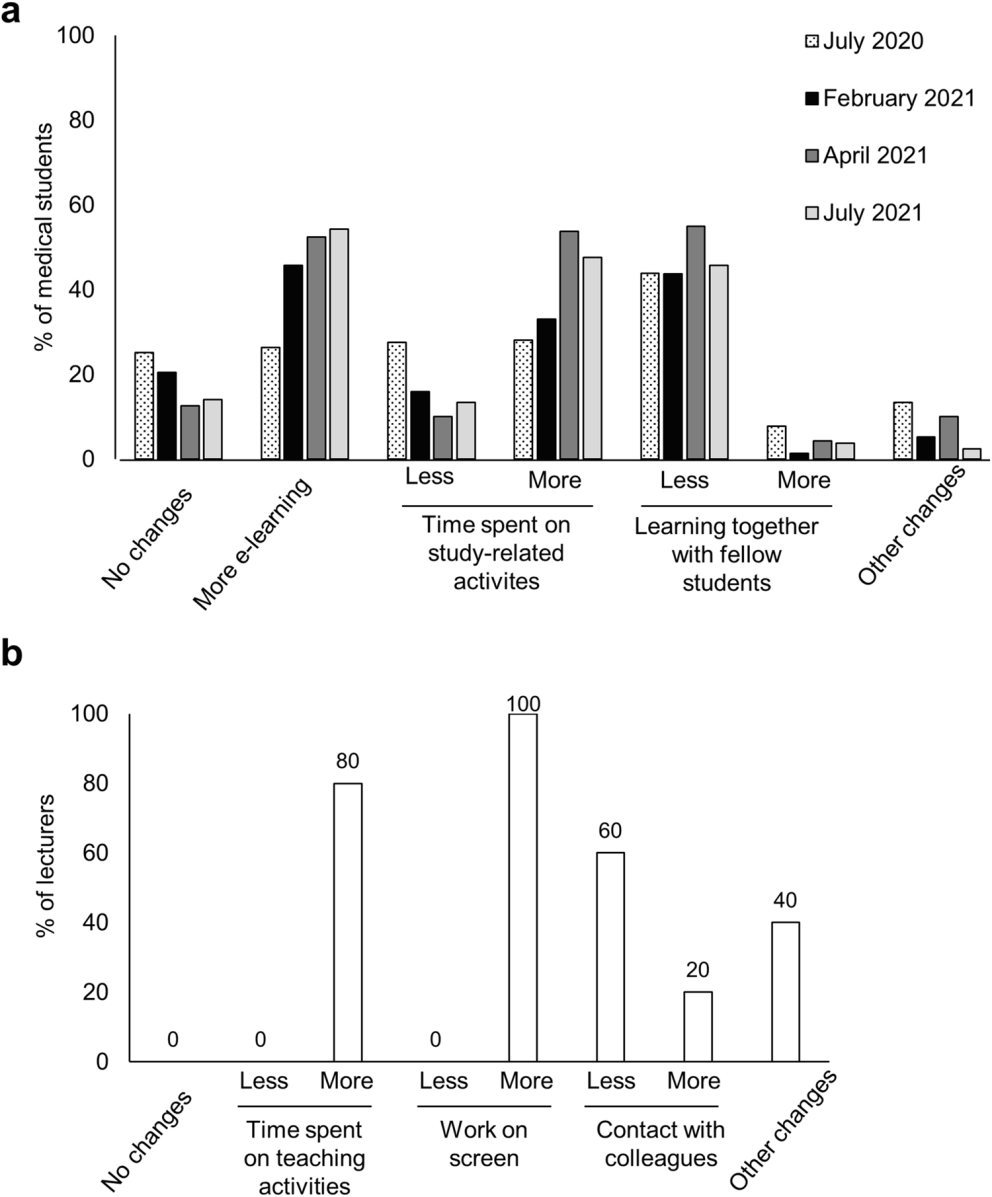
Changes in learning and teaching behaviors of medical students and university teachers during the COVID-19 pandemic. **a** Change in learning behaviors of medical students during the COVID-19 pandemic (% of respondents from July 2020 to July 2021), *n* = 763 respondents. **b** Change in teaching behaviors of university lecturers during the COVID-19 pandemic (% of respondents, questioned in December 2021), *n* = 5 respondents

**Fig. 5:**
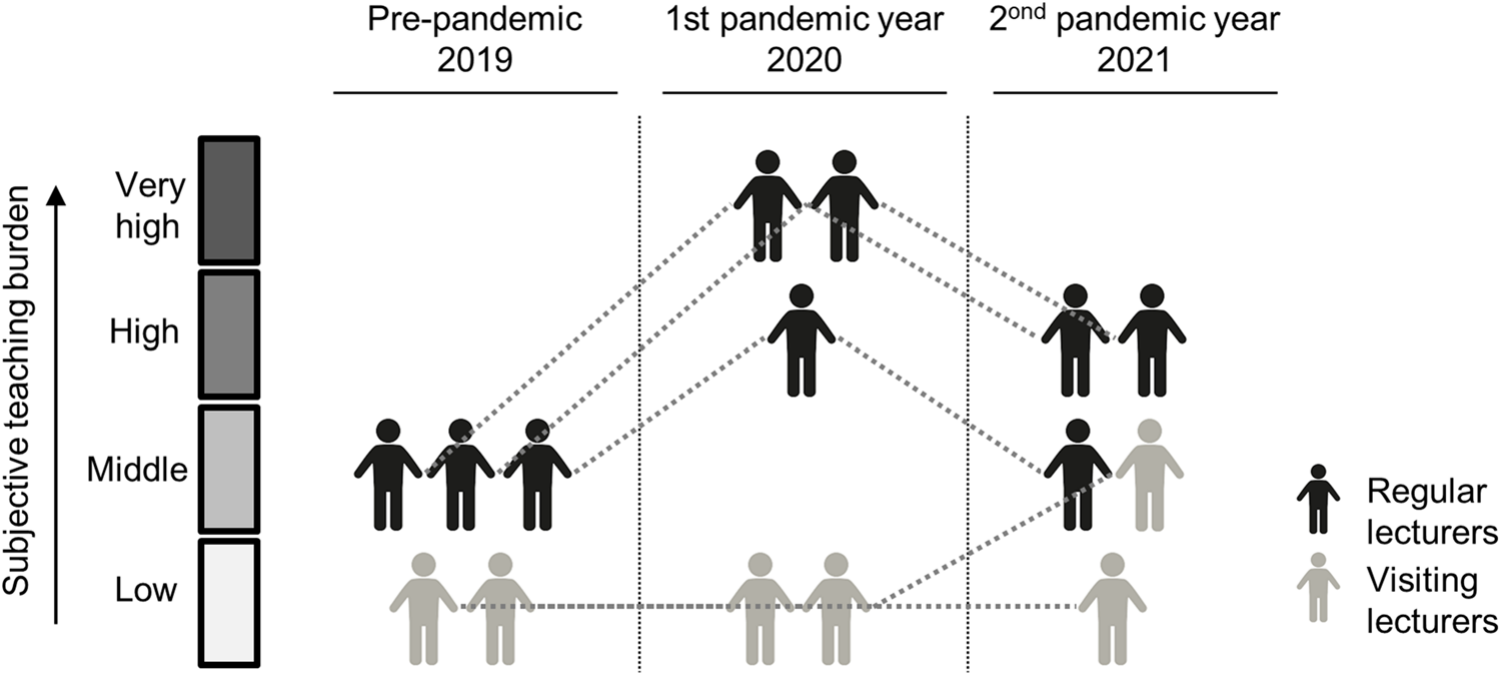
Subjective teaching–related burden indicated by university teachers in clinical pharmacology. Lecturers were asked for their subjective teaching burden during in pre-pandemic time (2019), in the 1st year of the COVID-19 pandemic (2021), and in the 2nd pandemic year. The graph presents each lecturer’s answers as stickman icons interconnected with a dashed line. Black icons stand for regular lecturers employed at the Institute of Clinical Pharmacology, whereas gray icons indicate visiting lecturers

Most students and lecturers stated that they spent more time on study or teaching-related activities, respectively, and had less contact with fellow students or colleagues in comparison to pre-pandemic times (Fig. 4). All teachers indicated that they would spend more time on the screen than before the pandemic (Fig. 4b). Furthermore, the subjective burden for 4 of 5 lecturers increased during the pandemic: Whereas all lecturers considered their teaching burden as low or middle in the pre-pandemic time, 3 of 5 lecturers indicated a high or even very high teaching-related burden in the first year of the pandemic (Fig. 5). In the 2nd pandemic year, the subjective burden decreased, but was still considered high by 2 lecturers and not back to pre-pandemic levels in 3 of 5 teachers.

### Correlations with exam grading

To identify determinants of learning success, the survey data were correlated with gradings in the written exams of clinical pharmacology (Table 3). In the first online semester (summer term 2020), the use of paper-based learning scripts was correlated with better exam grades. No correlation with grade was found for any other learning materials. Age was inversely correlated with exam grading.

**Table 3:**
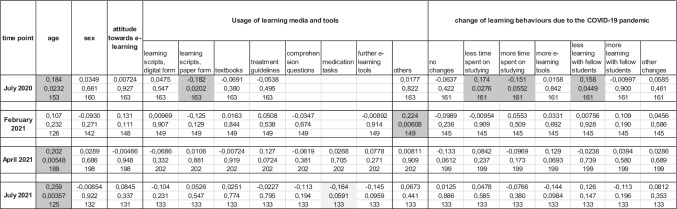
Correlations of exam grading as an indicator of learning success with learning behaviors and student characteristics. Spearman correlations were conducted using Sigma plot. Each field shows the correlation coefficient, *p*-value, and number of datasets (*n*) for the given correlation. Significant correlations are marked with gray color. Positive correlations indicate that the respective parameter was associated with higher (i.e., worse) exam grade, and vice versa

## Discussion

Our survey on medical education in clinical pharmacology at the Southern German University of Ulm exemplifies how learning and teaching formats and habits have changed during the COVID-19 pandemic. Even though this is not surprising, our survey unravels these changes in detail, and contrasts students’ and lecturers’ views. The data indicate a need for action to develop common goals for medical education in the near future.

### Traditional learning materials

Traditionally, studying at a university was associated with attending lectures and taking notes, spending long reading hours at university libraries, and searching thick folders of hard copies. Likewise, most surveyed students used paper-based and digital learning scripts in parallel in the pre-pandemic winter term and also in the first semester during the pandemic, when online teaching at the Institute of Clinical Pharmacology was at its very beginning (Fig. 1a). However, due to the pandemic, universities were completely closed for months and students did not have access to libraries and printing shops.

Furthermore, the increasing establishment of online education significantly changed teaching and learning behaviors (Fig. 4). According to a survey in 2015, more than 99% of students had internet access at home and German higher education students were equipped with 5 different digital devices on average, although these devices were not primarily used within their studies (Zawacki-Richter et al. 2015). Therefore, the step from classroom to online education could be rapidly achieved due to pre-existing digital infrastructure. This might explain the clear drop in the use of paper-based scripts during subsequent terms in 2021 within our study (Fig. 1a).

Interestingly, we observed the same trend with textbooks, although the most common textbooks in clinical pharmacology are available as e-books. Even more, a decreasing number of students were reading original versions of treatment guidelines, even though these are published online. It seems that students during the pandemic focused on the materials provided through the learning management system, and did not seek further and more comprehensive information.

### Learning management systems: opportunities and limitations

At Ulm University, the learning platform and course management system “moodle” has been established for many years to provide learning materials and further information on every single course and subject. However, teaching has so far mainly taken place in the classroom in direct interaction with individual lecturers, who clarified (exam-) relevant questions and provided further information. It is common practice that lecturers recommend certain textbooks and additional sources of information. With the pandemic, the learning management system got the only source of study-related information. Lecturers placed all teaching and learning materials and further information there, and questions were answered via integrated forums. Consequently, it seems that learning behavior lost its diversity and was reduced to the materials provided via the learning management system, which might be the most efficient way to prepare for the corresponding exams.

However, regarding a comprehensive medical education in clinical pharmacology, searching for reliable information (e.g., in treatment guidelines and textbooks) is a crucial aspect to keep oneself “up-to-date” on current therapeutic standards and changes. Providing all course-relevant information in one place (like a learning management system) is convenient and efficient, but should be complemented by the acquisition of knowledge on how physicians can gather evidence-based information on pharmacotherapy during their working life. Additional and precisely designed courses might close this gap: For example, at Hannover Medical School, an elective course for the acquisition of data literacy was implemented online in 2020 (Behrends et al. 2020). The course design was based on an online module provided by the HiGHmeducation consortium. This body is part of the Medical Informatics Initiative and funded by the German Federal Ministry of Education and Research (BMBF) (Witte et al. 2020). It aims to build up a didactical framework for online teaching in order to address the increasing needs. The further propagation and usage of such modules might be a valuable help to adapt teaching offers to current needs.

### Attitude towards e-learning

In our study, 80% of the lecturers saw a high benefit in learning scripts and classroom teaching, i.e., traditional methods. Any online methods were rated as beneficial by no more than 1 lecturer (20%). Of note, newly introduced or developed methods during the pandemic are associated with additional efforts and workload for most lecturers (Fig. 5). In addition, some lecturers faced technical or internet-related problems in the early time of the pandemic when remote access and online classes had to be established. This might bias lecturers’ evaluation of the utility of these formats and materials, and might also contribute to a rather skeptical attitude towards e-learning. Students appeared very open to e-learning tools in general, and the majority had a positive perception of the newly introduced digital comprehension tests and medication tasks.

### Benefit evaluation

Still, this did not curtail the utility evaluation of traditional learning scripts which was still rated high by students as well. Live-and-scheduled online lectures were rated less valuable by both, students and lecturers, whereas only students saw a benefit from time-independent online lectures. Indeed, any online lecture formats lack various aspects of interaction and (non-verbal) communication. However, lecture videos have the advantage of time-independent usability and repeatability as required, whereas live-and-scheduled formats do not offer any additional advantage. This result is consistent with a survey among German medical students at the University of Leipzig in September 2020: Here, more than 90% of the participating students stated that traditional classroom lectures should be permanently replaced by digital alternatives like audio or video podcasts (Hempel et al. 2021). Another survey among medical students at the University of Göttingen compared digital teaching in psychiatry during the pandemic to classroom teaching before the pandemic and did not see a loss of quality in terms of individual learning effect and processing of learning goals (Besse et al. 2022). In clinical pharmacology, learning goals contain mainly theoretical skills, and a mixture of online and classroom elements appears didactically appropriate.

### Changes of learning and teaching during the pandemic

The pandemic-related measures transformed working, living, and communication on a global level. The sudden and unforeseen transition from face-to-face to online education went along with a lot of changes and challenges within the educational system. In our study, students and teachers reported a higher working load and screen time or e-learning activities, respectively (Fig. 4). Despite significant advantages and new possibilities, online education comes along with difficulties as well (Garcia-Morales et al. 2021). For example, working and studying at home lacks social exchange and active academic involvement. Likewise, the participants of our study stated less contacts with colleagues or fellow students (Fig. 4).

All over the world, medical education is challenging and often perceived as stressful, and medical students are at higher risk for anxiety disorders and depression in comparison to the general population (Moir et al. 2018; Quek et al. 2019). Social distancing, fear of being infected, and worries about family members and about the future generate additional stress and might negatively impact mental health of students and employees. At Cyprus University Medical School, the impact of digital learning during the pandemic on students’ mental health was assessed (Zis et al. 2021): Whereas burnout rates did not significantly differ in comparison to a pre-pandemic cohort, mental health scores decreased, and emotional exhaustion was elevated in final-year students. Therefore, in addition to feasibility and utility, social- and health-related aspects should be taken into account when new teaching and learning formats are developed.

Beyond pandemic-related changes, the curriculum of medical studies should account for the digitization of the German healthcare sector. Although in Germany this process is running slow in comparison to other countries as shown by the 2018 report of the Bertelsmann Foundation (Thiel et al. 2018), an interview study including 30 faculty representatives revealed that most faculty representatives think the digitization of the healthcare sector should be given higher priority in the curriculum (Neumann et al. 2021).

### Limitations and strengths

Pandemic-related measures at German universities are regulated by each federal state, which limits the transferability of the data to other universities across the country. Furthermore, the survey was a single-center study and the results may be influenced by various location-specific circumstances. For example, there was a staff turnover coinciding with the pandemic when an experienced clinical pharmacologist and full-time professor left the institute in early 2020. Therefore, in addition to pandemic-related changes, the personnel situation was probably contributing to a higher teaching load of the remaining lecturers.

A particular strength of this survey is the fact that it includes a pre-pandemic cohort as a comparison group because the study was initiated in 2019 when the imminent pandemic was not foreseeable.

The validity of most online surveys is limited by a high degree of response bias. Therefore, we decided to continue paper-based surveying even during the pandemic. This was feasible because the written exam in clinical pharmacology was performed under strict measures and in presence even during the lockdown period. Remarkably, the response rates among students in all questioned cohorts were very high (Table 1). As another indicator of data validity, we compared the exam grading of respondents to the overall cohort and found only marginal differences (Table 1).

## Conclusions

This survey demonstrates how the pandemic-related digital transformation has changed learning and teaching formats and habits. Although new digital formats are well accepted by most students, their implementation requires additional efforts and increases study and teaching load. Our study highlights the conflicting attitudes and perceptions of students and teachers. It seems important to strike a balance between the future visions and needs of either side. In addition, medical students should be prepared for the digitization of the German healthcare sector. However, beyond feasibility and utility, social- and health-related aspects should be taken into account when new teaching and learning formats are developed.

## Supplementary Information

Below is the link to the electronic supplementary material.

Supplementary file1 (DOCX 16 KB)

## Data Availability

The datasets used and/or analyzed during the current study are available from the corresponding author on reasonable request.

## Declarations

### Author information

The authors have long-standing experience and take responsibility in medical education in pharmacology.
