# Supplementary material for: COVID-19 pandemic–related adaptations of medical education in clinical pharmacology — impact on students and lecturers at a German university
Source: Naunyn Schmiedebergs Arch Pharmacol. 2022 Mar 18;395(6):681–90. doi: 10.1007/s00210-022-02225-3 (PMC8930485; doi:10.1007/s00210-022-02225-3)
Supplement: Supplementary file 1 — Supplementary file1 (DOCX 16 KB) [file 210_2022_2225_MOESM1_ESM.docx]

| Curriculum - Clinical Pharmacology and Pharmacotherapy | |
| --- | --- |
| Format | Semester 7: Lecture, 2 semester periods per week  Semester 8/9: Seminar, 10.3 semester periods per week (8 groups) |
| Content | The **lecture** in clinical pharmacology is intended to convey basic elements of clinical pharmacology and evidence-based medicine. Furthermore, it covers specific pharmacotherapeutic concepts and algorithms to treat important diseases according to current medical guidelines.  In **seminars**, medication regimens for authentic patient cases are developed, discussed and optimized on the basis of clinical pharmacotherapeutic guidelines. |
| Examination | Written examination – multiple choice questions, max. 40 points  Oral examination – max. 20 points  Pass limit: 60%  Exam Failure: 2 possible retries |

Supplementary table S1: Curriculum in Clinical Pharmacology and Pharmacotherapy at Ulm University
